# Are Vitamin D_3_ Tablets and Oil Drops Equally Effective in Raising S-25-Hydroxyvitamin D Concentrations? A Post-Hoc Analysis of an Observational Study on Immunodeficient Patients

**DOI:** 10.3390/nu12051230

**Published:** 2020-04-26

**Authors:** Maria Helde Frankling, Anna-Carin Norlin, Susanne Hansen, Emilie Wahren Borgström, Peter Bergman, Linda Björkhem-Bergman

**Affiliations:** 1 Department of Neurobiology, Care Sciences and Society (NVS), Division of Clinical Geriatrics, Blickagången 16, Neo floor 7, SE-141 83 Huddinge, Sweden; linda.bjorkhem-bergman@ki.se; 2 ASIH Stockholm Södra, Långbro Park, Palliative Home Care and Hospice Ward, Bergtallsvägen 12, SE-125 59 Älvsjö, Sweden; 3 Department of Infectious Diseases, The Immunodeficiency Unit, Karolinska University Hospital Huddinge, SE-141 86 Stockholm, Sweden; anna-carin.norlin@sll.se (A.-C.N.); susanne.hansen@sll.se (S.H.); emilie.wahren-borgstrom@sll.se (E.W.B.); peter.bergman@ki.se (P.B.); 4 Department of Laboratory Medicine, Division of Clinical Microbiology, Karolinska Institutet, SE-141 86 Stockholm, Sweden; 5 Stockholms Sjukhem, Palliative Home Care and Hospice Ward, Mariebergsgatan 22, 112 19 Stockholm, Sweden

**Keywords:** vitamin D, Detremin (cholecalciferol), Divisun (cholecalciferol), cholecalciferol, supplementation, clinical trial, infections, antibiotics, oil drops, tablets, S-25-OHD

## Abstract

Background: Vitamin D_3_ supplements are available as tablets or oil drops, but there is no consensus as to whether either of these preparations is more effective than the other. Methods: We compared the effectiveness of tablets versus oil in raising S-25-hydroxyvitamin D (S-25-OHD) in plasma by re-analyzing data from a previously performed observational study in which immunodeficient patients with S-25-OHD concentrations <75 nmol/L were randomly prescribed vitamin D_3_ tablets (1600 IU/day) or vitamin D_3_ oil-drops (1500 IU/day) for twelve months. Tablets and oil were compared for the effect on S-25-OHD concentrations after 3–5 months and antibiotic use. Results: Data on S-25-OHD after ≥ 3 months was available for 137 patients treated with tablets and 69 with oil drops. Both groups exhibited a significant increase in S-25-OHD—oil-drops from 55 to 86 nmol/L and tablets from 52 to 87 nmol/L—with no difference between groups (*p* = 0.77). In a subgroup of patients without immunoglobulin replacement, vitamin D_3_ supplementation with oil drops (*n* = 34) but not with tablets (*n* = 60) resulted in significantly lower antibiotic administration (*p* < 0.001 and *p* = 0.58). Conclusion: Vitamin D_3_ supplementation with tablets and oil drops were equally efficient in raising S-25-OHD concentrations. Only oil drops caused a reduction in antibiotic consumption in immuno-deficient patients who did not receive immunoglobulin replacement.

## 1. Introduction

Vitamin D_3_, or cholecalciferol, is a compound synthesized endogenously from cholesterol. The synthesis takes place in the skin using energy from UVB radiation in sunlight. Vitamin D_3_ is hydroxylated into its active form 1,25-dihydroxyvitamin D (1,25-OHD) in a two-step hydroxylation process. The active form 1,25-OHD binds to the vitamin D receptor, which is present in many tissues throughout the body. Vitamin D plays an important role in calcium homeostasis, contributes to maintaining bone health, and modulates both innate and adaptive immune responses [1,2,3]. Vitamin D status of an individual is assessed by measuring 25-hydroxyvitamin D (25-OHD) concentrations in serum or plasma, which is relatively stable and has a longer half-life compared to the active metabolite 1,25-OHD [4]. Synthetically derived vitamin D_3_ (cholecalciferol) can be administered as tablets or oral drops dissolved in oil preparations. 

Observational studies have shown an association between low 25-OHD concentrations and increased susceptibility to respiratory tract infections [5,6]. Furthermore, increasing evidence suggests that vitamin D supplementation is beneficial to boost the immune system [3,7] through effects on both the innate immune system, by inducing the synthesis of antimicrobial peptides [8], and on the adaptive immune system, by regulating the T-cell response and cytokine levels [5]. A meta-analysis comprising 25 randomized controlled trials (RCTs) studying the effect of vitamin D_3_ supplementation on respiratory tract infections (RTIs) has shown significant protective effects of daily or weekly vitamin D_3_ supplementation on the frequency of respiratory tract infections. The effect was greater in patients with severe vitamin D deficiency [7]. 

One of the included RCTs was performed at the Immunodeficiency Unit at Karolinska University Hospital. It was comprised of 140 patients with selective IgA subclass deficiency, immunoglobulin (IgG) subclass deficiency, or common variable immune disorder (CVID) with frequent RTIs who were randomized to placebo or 4000 International Units (IU) of vitamin D_3_/day for 12 months. This study showed that vitamin D_3_-supplemented patients had a lower infectious score and fewer respiratory tract infections compared to the placebo group [9,10]. Based on these results, amongst others, the national guidelines for immunodeficient patients in Sweden now recommends analysis of the serum level of 25-OHD and supplementation with vitamin D_3_ 1500–1600 IU/day, allowing the physician to choose between the two cholecalciferol-containing products available on prescription in Swedish pharmacies. Patients are prescribed vitamin D_3_ as two Divisun tablets at 800 IU each or as Detremin, 3 drops at 500 IU/drop, yielding daily doses of 1600 or 1500 IU/day, respectively [11]. Previous studies have shown that vitamin D deficiency is rather common among immunodeficient patients, and that approximately half of such patients have 25-OHD<50 nmol/L [11,12,13]

In 2013, the clinical effects of the new guidelines were evaluated in a prospective, observational follow-up study comparing antibiotic use during a 12 month period before and after initiating supplementation in immunodeficient patients (*n* = 277) [11]. The results showed a significant reduction in the number of days on antibiotic treatment, a reduction in the number of prescriptions of antibiotics, and an increased number of entirely antibiotic-free patients. A subgroup analysis revealed that only patients without immunoglobulin replacement (*n* = 135) exhibited a significant benefit from the intervention. In total, *n* = 182 patients were treated with either vitamin D_3_ tablets (Divisun, 800 IU/tablet), and *n* = 95 patients were treated with oral oil drops (Detremin, 500 IU/drop) [11].

It is desirable for improving acceptability and compliance to offer different types of preparations of vitamin D_3_. Today, there is no consensus whether any of the different vitamin D_3_ preparations for oral use would be better than the other in raising serum concentrations of 25-OHD. Indeed, comparisons between the effects of supplementation with vitamin D_3_ tablets versus oral oil drops in healthy adult patients are scarce, and in the few studies performed no administration method has been shown to be superior [14,15,16,17].

The aim of this study was therefore to test the hypothesis that equivalent doses of vitamin D_3_ in tablets and in oil are equally effective in raising S-25-hydroxyvitamin D (S-25-OHD). As a secondary endpoint, we analyzed possible differences in antibiotic consumption between the group treated with oil drops and the group treated with tablets. To this end, we re-analyzed the existing data set from our previously performed observational study from 2013, where immunodeficient patients were supplemented with either vitamin D_3_ tablets or oil drops [11].

## 2. Materials and Methods

We performed a post-hoc analysis using an existing data set from an observational study recruiting between March and October 2013 and performed at the Immunodeficiency unit at Karolinska University hospital [11]. In the original study, 277 immunodeficient patients who fulfilled the single inclusion criterion of S-25-OHD <75nmol/L were supplemented with Vitamin D_3_ administered as tablets or oil drops, in doses previously specified [11]. Patients who were already on treatment with cholecalciferol or active vitamin D (Etalpha), as well as patients with tuberculosis or sarcoidosis were excluded. A majority of patients in the original cohort were women (*n* = 175, 63%), and the median age was 55 years (range 81–90). Selective IgA deficiency was present in *n* = 44 patients, and IgG subclass deficiency and CVID in *n* = 80 and *n* = 52 patients, respectively. A total of 135 patients received IgG replacement therapy (all of the patients with CVID, almost half of patients with IgG subclass deficiency, and 10% of patients with selective IgA deficiency). Compliance was monitored by asking patients at every visit and recording the answer in the patients chart [11]. 

In order not to favor any specific drug company, the intention was to prescribe tablets and oral drops to alternate patients included. Actual prescriptions did not entirely adhere to this intention; instead, there was a 2:1 relationship between patients prescribed tablets and patients prescribed oral drops in the cohort. A retrospective analysis showed that the reason for the skewed prescription pattern was patient preference (many patients preferred tablets instead of oil, which was accepted in the study). 

**Figure 1:**
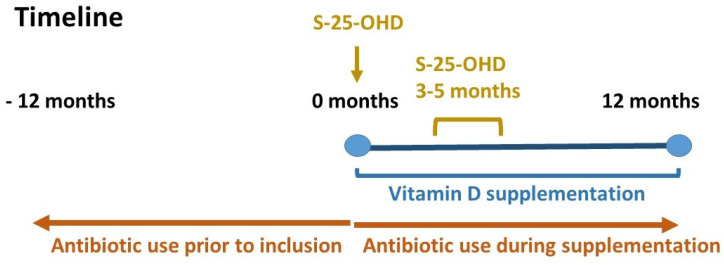
Timeline.

In the original study, the intention was to measure S-25-OHD at inclusion and at every visit to the Immunodeficiency Unit during the follow-up period—four visits over one year. However, few patients had their S-25-OHD -concentrations measured regularly after the first follow-up visit. In this analysis, we therefore compared S-25-OHD at baseline with the serum value at the first follow-up visit, which took place after three to five months of supplementation. Only patients with recorded measurements of S-25-OHD both before starting intervention and at their first visit to the Immunodeficiency Unit during intervention were included in the analysis. The Swedish Prescribed Drug Registry provided data on prescriptions of oral antibiotics. Data on the number of days on prescribed antibiotics during the 12 month periods before and after inclusion in the study were retrieved from the registry and are presented as both number of days on antibiotics and number of prescriptions (Figure 1) 

The original study was approved by the regional Ethical Review Board at Karolinska Institutet, Stockholm, Sweden (dnr 2013/2244-31/1). The Swedish Medical Product Agency has stated that criteria for a “non-interventional clinical trial” were fulfilled. Written informed consent was obtained from all participants before inclusion and the study was conducted in accordance with the Declaration of Helsinki.

**Figure 2:**
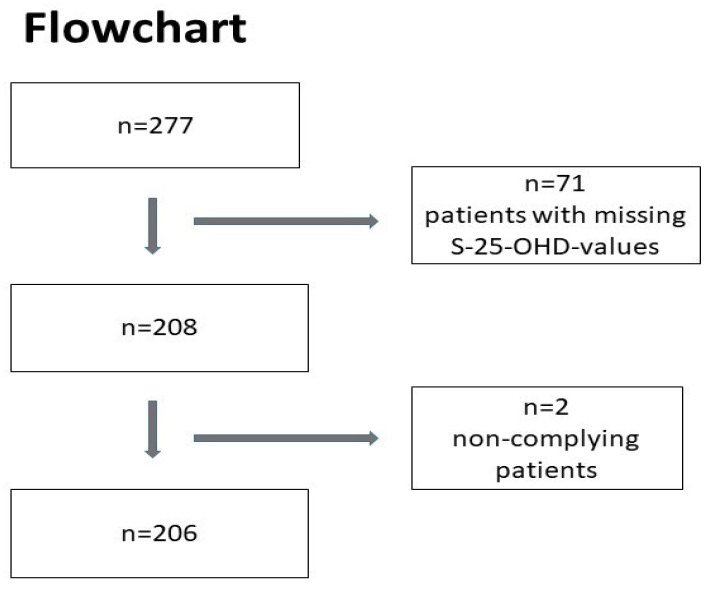
Flowchart.

**Figure 3:**
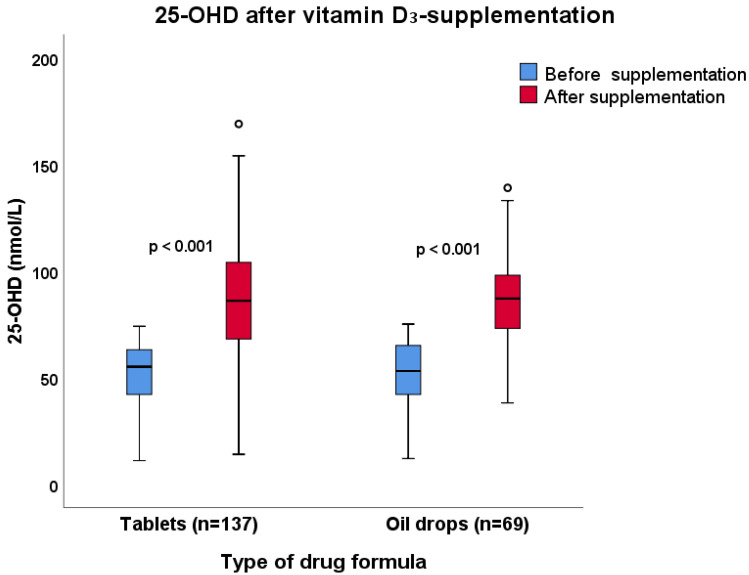
Outcome for S-25-hydroxyvitamin D (S-25-OHD) concentrations in immunodeficient patients receiving vitamin D_3_ supplementation as oil drops (1500 IE/day) or as tablets (1600 IE/day) for 3–5 months. Median S-25-OHD increased from 55 to 86 nmol/L in the group taking oral drops and from 53 to 87 nmol/L in the group taking tablets. There was no difference in the increase in S-25-OHD from baseline between the groups (Mann–Whitney U test, *p* = 0.77).

Statistical analysis was performed using SPSS 26. Since S-25-OHD concentrations were not normally distributed, non-parametric tests were used. For comparisons between groups regarding baseline demographic data, Fischer’s exact was used for dichotomous variables—namely, gender, presence of IgG replacement, antibiotic use vs no antibiotic use, and Mann–Whitney U for continuous variables (age, S-25-OHD concentration, number of prescriptions, and number of days on antibiotics/year), as shown in Table 1. A Wilcoxon matched pairs signed-rank test was used for the comparison of the continuous variables S-25-OHD concentration, number of prescriptions, and number of days on antibiotics/year before and after supplementation, as shown in Table 2. McNemar’s test was used to analyze the variable “no antibiotic use” before and after intervention. Mann–Whitney U was used to calculate the difference in the increase of S-25-OHD concentration between the tablet and oil drop groups (Figure 1). A Wilcoxon matched pairs signed-rank test was used in the subgroup analysis comparing immunodeficient patients with immunoglobulin (IgG) replacement to patients without IgG replacement regarding the effect of vitamin D supplementation (Figure 2 and Figure 3). In the tables, the median values, interquartile range (IQR), and minimum and maximum values for continuous variables are presented.

## 3. Results

The cohort was reduced to *n* = 137 patients treated with tablets and *n* = 69 patients treated with oral drops, after excluding patients with missing S-25-OHD concentration values, as well as two patients in the oil drops group who unexpectedly exhibited significantly decreased concentrations of S-25-OHD after the intervention (−31 and −42 nmol/L), and who probably failed to take the prescribed supplementation (Figure 2)

### 3.1. Baseline Demography

Most included patients were women (*n* = 129; 63%) and the median age was 56 years (IQR 29, range 19–88). Median serum concentrations of S-25-OHD before intervention were slightly above 50 nmol/L in both groups, with a wide range in serum concentrations, including 11 patients with severe vitamin D deficiency with S-25-OHD ≤30 nmol/L. The groups were well matched at baseline, without any statistically significant differences (Table 1).

### 3.2. S-25-OHD Concentrations 

At the first visit after study inclusion (3–5 months post-inclusion), there was a significant increase in median S-25-OHD (*p* < 0.001) in both groups, from 55 to 86 nmol/L in the group taking oral drops and from 53 to 87 nmol/L in the group taking tablets. There was no statistically significant difference in the increase in S-25-OHD from the baseline between the two groups (*p* = 0.77) (Figure 3).

### 3.3. Antibiotic Consumption

The number of patients who did not receive antibiotics (“antibiotic-free”) increased from 10 to 21 (*p* = 0.03) in the group taking oil drops when comparing the 12 month period prior to the intervention to the 12 month period with supplementation. The number of “antibiotic free” patients increased in the group taking tablets as well, from *n* = 22 to *n* = 32, but this change was not statistically significant (*p* = 0.13). In the group taking oil drops, both the number of prescriptions (*p* = 0.003) and the number of days with antibiotics (*p* = 0.003) were significantly reduced during supplementation compared to the previous year without supplementation (Table 2).

### 3.4. Subgroup Analysis

**Figure 4:**
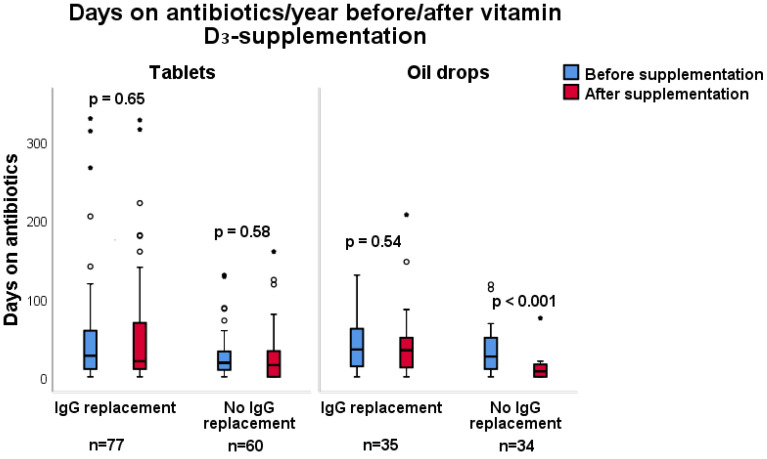
Subgroup analysis comparing immunodeficient patients with immunoglobulin (IgG) replacement to patients without IgG replacement, regarding the effect of vitamin D_3_ supplementation as oil drops (1500 IE/day) or as tablets (1600 IE/day) for one year and antibiotic requirements, expressed as number of days on antibiotics. Patients are their own controls, and the 12 month period before supplementation is compared to the 12 month supplementation period. In patients without immunoglobulin replacement therapy who were supplemented with oil drops (*n* = 34), the median number of days on antibiotics decreased from 26 (0–118) to 7 (0–75), *p* < 0.001 (Wilcoxon matched pairs signed rank test).

**Figure 5:**
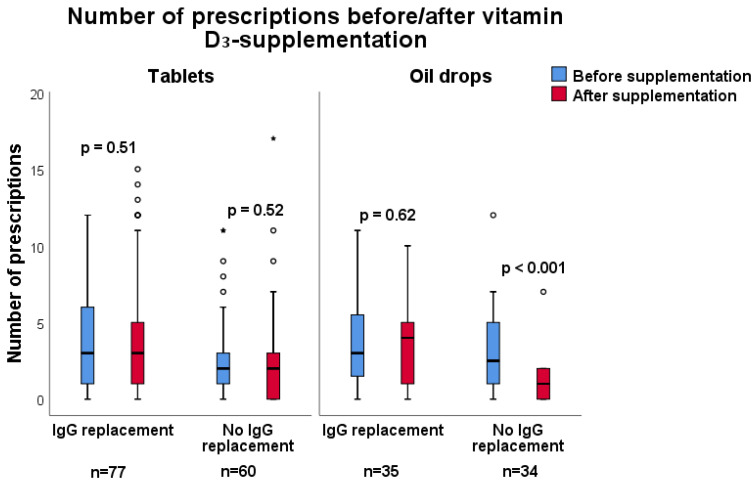
Subgroup analysis comparing immunodeficient patients with immunoglobulin (IgG) replacement to patients without IgG replacement regarding the effect of vitamin D_3_ supplementation as oil drops (1500 IE/day) or as tablets (1600 IE/day) for one year and antibiotic requirements, expressed as number of prescriptions of antibiotics. Patients are their own controls, and the 12 month period before supplementation is compared to the 12 month supplementation period. In patients supplemented with vitamin D_3_ oil drops who did not receive IgG replacement, the median number of prescriptions decreased from 3.1 (0–12), to 1 (0–7), *p* < 0.001 (Wilcoxon matched pairs signed-rank test).

When comparing the effects of vitamin D_3_ supplementation administered as tablets vs oral drops in the patients without immunoglobulin replacement therapy, significant results regarding number of prescriptions and days on antibiotics were only observed in the group taking oil drops (Figure 3 and Figure 4). In patients without immunoglobulin replacement therapy who were supplemented with oil drops (*n* = 34), the number of days on antibiotics decreased from 26 to 7 (*p* < 0.001), and the number of prescriptions decreased from 2.5 to 1 (*p* < 0.001). In contrast, patients taking tablets did not exhibit a significant change in the number of days on antibiotics (Figure 4 and Figure 5). Significant results are highlighted in bold. 

## 4. Discussion

The primary finding in this study was that vitamin D oil drops and tablets were equally effective in raising S-25-OHD concentrations in the circulation. This finding supports the notion that prescribers and patients can choose the formulation they prefer for the individual patient in order to raise S-25-OHD levels in the circulation. We also observed that the beneficial effect on antibiotic consumption was restricted to those taking oil drops and to the subgroup without immunoglobulin replacement. This finding was unexpected and potentially important for vitamin D_3_ supplementation strategies in different patient groups. 

The effect of vitamin D_3_ supplementation on the increase of S-25-OHD concentrations in this study is well in line with results from previous studies. The Institute of Medicine (IOM) concludes in their review on dietary reference intakes that 40 IU vitamin D_3_ raises S-25-OHD concentration by approximately 1 nmol/L, which means that an oral intake of 1500–1600 IU/day would raise S-25-OHD concentrations by 37.5–40 nmol/L [18]. Here, we found a mean increase of 31–34 nmol/L, which was slightly lower than anticipated. However, there may be several reasons for this observation, and lack of compliance is one important factor to consider. 

A prospective, randomized study on 55 healthy adults in Norway showed similar increases in S-25-OHD after four weeks of supplementation with multivitamin tablets or fish oil capsules containing 400 IU vitamin D_3_. A mean increase of 35.8 nmol/L (95% CI 30.9–40.8) was observed in the multivitamin group, whereas the oil capsule group exhibited an increase of 32.3 nmol/L (95% CI 27.3–37.4) [16,17]. In patient populations, the matter has been studied in some cohorts of patients with cystic fibrosis. Since pancreatic insufficiency causes fat malabsorption in patients with cystic fibrosis, it is theoretically possible that powder-based vehicles may be more effective than oil preparations in this patient group. Results from these studies do not, however, suggest that there is likely to be any clinically significant difference between the use of tablets or oil preparations [14,15,19].

It should be noted, however, that previous studies have mainly focused on S-25-OHD concentrations in the circulation and not on clinical outcomes. Here we observed that only oil drops caused a significant reduction in antibiotic consumption among those patients without immunoglobulin replacement therapy. It is possible that this finding could have a mechanistic basis, since oil emulsions might be better absorbed in the gut than tablets [20]. In this scenario, oil preparations would reach the liver faster via the portal vein, and could thus cause a more efficient downstream effect on target cells. It is important to note that S-25-OHD concentration, although widely used as a marker for vitamin D status, is only is a rough estimate of the vitamin D metabolism. In fact, the most important effect of vitamin D occurs in the microenvironment, where a paracrine loop is active and locally available 25-OHD is taken up by cells for intracellular conversion to the active form, 1,25-OHD vitamin D [21,22,23]. Active vitamin D binds to its cognate receptor (VDR), and then the complex is translocated to the nucleus and binds to vitamin D responsive elements, which activate a large set of target genes [24]. Given this complex activation pathway for vitamin D, it is possible that there are effects not directly related to S-25-OHD concentrations in the circulation [25]. In addition, active vitamin D may have non-genomic effects mediated via VDR, located in the caveolae in plasma-membranes [26]. This pathway mediates a rapid increase in intracellular calcium release, which leads to downstream events, such as increased exocytosis and preventions of apoptosis [27]. This is an interesting hypothesis to pursue further, but due to the low number of patients in this study, the present results should be interpreted with caution.

This study has several strengths. It is to our knowledge the first study where tablet and oil preparations of vitamin D_3_ are compared in adult patients without cystic fibrosis. Another strength is that this is the first study where not only S-25-OHD concentration is measured, but also a clinically more relevant endpoint (antibiotic consumption). The observation that only oil drops caused a beneficial effect is somewhat unexpected, but nevertheless important. It could serve as a starting point for additional studies comparing tablets and oil preparations in relation to other outcomes (musculoskeletal, inflammatory, cardiovascular). Preferably, those studies should include measurements of serum markers of inflammation.

However, the study also has some limitations—mainly related to the design—that need to be addressed. First, the time between measurement of S-25-OHD at baseline and follow-up varied between subjects from 3–5 months after starting supplementation. In a review from 2015, it was suggested that the maximum effect of vitamin D_3_ supplementation occurred after 3 months, and one study suggested that a duration of six months was even better [28]. However, it should be noted that even though S-25-OHD concentrations were recorded after 3–5 months post-inclusion, the clinical outcome (antibiotic consumption) was measured after 12 months. This could explain the fact that no difference was observed in S-25-OHD concentrations between those taking tablets or oil, but still an effect was found on the clinical outcome. Second, there is a risk of selection bias, since there was no bona fide randomization in the study. Rather, a quasi-randomization scheme was applied, where every second patient should have had tablets and every second should have had oil. However, since several doctors recruited patients and patients’ own preferences were accepted, the system could not achieve the planned randomization. Still, the groups were well-balanced at the start of intervention, as shown in Table 1, which lends credibility to the results. Furthermore, seasonal variations in S-25-OHD due to sun exposure were not taken into account in this study. Earlier studies from 59° N have shown baseline values in the winter at 44 nmol/L and maximum concentrations in the summer at 59 nmol/L [29]. In this study, patients were included between March and October, and follow-up measurements were collected from June to February. There is a thus a risk that the concentrations of S-25-OHD were confounded by these seasonal variations. However, the fact that the increase in S-25-OHD concentration was well in line with the expected values, as stated by the Institute of Medicine, allows us to believe that the increase in S-25-OHD during the summer and decrease during the winter was well-balanced in the studied population [18]. Dietary intake of vitamin D was not recorded in this study, adding yet more uncertainty to the results. A final problem with the study relates to the generalizability of the results. The patients in this study exhibited various immunological disorders, mainly antibody deficiencies (IgA- and IgG-subclass deficiency), or CVID, but also a group that was classified as “increased susceptibility to infections” after a thorough immunological investigation. Thus, the results cannot be extrapolated to the general population, and further studies are needed to confirm or refute these hypothesis-generating results in other populations.

## 5. Conclusions

In conclusion, our results show that vitamin D administered as oil drops was equally effective as powdered tablets in raising S-25-OHD concentrations. This is an important finding with clinical implications. It means that doctors and patients can choose the type of preparation (tablet or oil) that suits the individual patient best. This will increase compliance and ultimately improve general health in the target population. Unexpectedly, we observed that oil drops were more effective than tablets in reducing antibiotic use, specifically in the subgroup of patients without immunoglobulin replacement. However, due to the low number of patients in this study and the subgroups, the results must be interpreted with caution. At this point, the data does not support the use of oil drops over tablets in patients with specific primary immunodeficiency and larger, preferably randomized and placebo-controlled studies are warranted before firm conclusions can be drawn.

## Figures and Tables

**Table 1:** Baseline demographics of the study population of immunodeficient patients who had been receiving vitamin D_3_ supplementation as oil drops (1500 IE/day) or as tablets (1600 IE/day) for one year at the time of inclusion.

Variable	Oil-Drops *n* = 69	Tablets *n* = 137	*p*-Value
Gender, *n* (%)			
Men	28 (41)	49 (36)	0.5 ^a^
Women	41 (59)	88 (64)	
Age, years Median (IQR) Mean–max	54 (36–63) 22–83	57 (40–69) 19–88	0.62 ^b^
S-25-OHD, nmol/L Median (IQR) Min–max	55 (42–63) 12–75	53 (42–66) 11–74	0.90 ^a^
IgG-replacement, *n* (%)	35 (51)	77 (56)	0.46 ^a^
No antibiotic use, *n* (%)	10 (15)	22 (16)	0.77 ^a^
Number of prescriptions Median (IQR) Min–max	3 (1–5) 0–12	2 (1–5) 0–12	0.40 ^b^
Days of antibiotics /year Median (IQR) Min–max	27 (10–52) 0–130	20 (10–48) 0–330	0.30 ^b^

^a^ Fisher’s test, ^b^ Mann–Whitney U test, S-25-OHD: S-25-hydroxyvitamin D; IQR: interquartile range; IgG: immunoglobulin.

**Table 2:** Outcome for S-25-hydroxyvitamin D (S-25-OHD) concentrations after 3 months and antibiotic consumption after 12 months in immunodeficient patients receiving vitamin D_3_ supplementation as oil drops (1500 IE/day) or as tablets (1600 IE/day) for one year.

Characteristic	Before Vitamin D_3_ Supplementation	After Vitamin D_3_ Supplementation	*p*-Value
S-25-OHD, ng/L			
Oil drops (*n* = 69) Median (IQR) Min–max	55 (42–63) 12–75	38–139 86 (68–104) 38–139	**<0.001** ^a^
Tablets (*n* = 137) Median (IQR) Min–max	11–74 53 (42–66) 11–74	87 (73–98) 14–169	**0.001** ^a^
Antibiotic free *n* (%)			
Oil drops (*n* = 69)	10 (14)	21 (30)	**0.03** ^b^
Tablets (*n* = 137)	22 (16)	32 (23)	0.13 ^b^
Number of prescriptions			
Oil drops (*n* = 69) Median (IQR) Min–max	3 (1–5) 0–12	2 (0–4) 0–10	**0.003** ^a^
Tablets (*n* = 137) Median (IQR) Min–max	2 (0–12) 0–12	2 (0–17) 0–17	0.35 ^a^
Days of antibiotics/year			
Oil drops (*n* = 69) Median (IQR) Min–max	27 (10–52) 0–130	15 (7–46) 0–207	**0.003** ^a^
Tablets (*n* = 137) Median (IQR) Min–max	20 (1–48) 0–330	19 (0–328) 0–328	0.43 ^a^

^a^ Wilcoxon matched pairs signed-rank test; ^b^ McNemar’s test. Bold numbers indicate significant results.
